# What do patients with substance use disorders know about their medication? A cross-sectional interview-based study

**DOI:** 10.3389/fpsyt.2025.1556920

**Published:** 2025-04-10

**Authors:** Johannes Heck, Melanie Dubaschewski, Olaf Krause, Stefan Bleich, Martin Schulze Westhoff, Benjamin Krichevsky, Alexander Glahn, Sebastian Schröder

**Affiliations:** ^1^ Institute for Clinical Pharmacology, Hannover Medical School, Hannover, Germany; ^2^ Department of Psychiatry, Social Psychiatry and Psychotherapy, Hannover Medical School, Hannover, Germany; ^3^ Institute for General Practice and Palliative Care, Hannover Medical School, Hannover, Germany; ^4^ Center for Medicine of the Elderly, DIAKOVERE Henriettenstift, Hannover, Germany

**Keywords:** medication knowledge, substance use disorder, alcohol use disorder, drug safety, medication plan

## Abstract

**Purpose:**

This study investigates the medication knowledge of patients with substance use disorders (SUDs) treated at a psychiatric clinic in northern Germany, aiming to identify gaps in understanding and to enhance patient safety, particularly concerning ATC group A drugs.

**Setting:**

The study was conducted in the Department of Psychiatry, Social Psychiatry, and Psychotherapy at Hannover Medical School, Germany.

**Design:**

A cross-sectional, interview-based study using a convenience sample of 100 patients was conducted between March 2023 and April 2024.

**Participants:**

The cohort included patients with SUDs who had been hospitalized for at least 72 hours, regularly took at least one medication in addition to withdrawal drugs, and who displayed no cognitive impairments. Participants had a median age of 46.5 years; 62% were male.

**Intervention:**

Patients were interviewed using a customized questionnaire addressing knowledge of drug name, indication, dosage, and frequency of application. The questionnaire also assessed the sources of medication knowledge and patient opinions on their medication regimen.

**Primary and secondary outcome measures:**

The primary outcome was the average medication knowledge score (range 0–6). Secondary measures included differences in knowledge across drug groups, sources of information, and demographic influences.

**Results:**

The median medication knowledge score was 3.8 out of 6. Knowledge was significantly lower for ATC group A drugs compared to groups B, C, and N (p < 0.001). No significant differences were observed between men and women nor between age groups. Hospital physicians were the primary information source for 40% of patients. Most participants (84%) considered their medication regimen adequate.

**Conclusion:**

Patients with SUDs demonstrated suboptimal medication knowledge, particularly regarding ATC group A drugs. Future strategies should prioritize patient education and enhanced physician engagement to improve understanding and adherence, ultimately fostering better therapeutic outcomes.

## Introduction

Substance use accounts for 5.5% of the global burden of disease (1). The incidence of alcohol and drug use disorders is increasing internationally, although there are significant regional differences (2). In addition to social consequences such as stigma, people with substance-related addiction disorders face a range of somatic risks, including cardiovascular, liver and pancreatic diseases (3–6). In addition, psychiatric comorbidities, including anxiety disorders, depression and attention deficit hyperactivity disorder, are highly prevalent among people with substance use disorders (SUDs) (7–9). The presence of multiple diagnoses often requires pharmacotherapy, leading to frequent polypharmacy among people with substance use disorders (10, 11).

Polypharmacy is associated with a lack of knowledge about medications, which can lead to reduced adherence to treatment and suboptimal treatment outcomes, especially in chronic diseases (12–14).

Inadequate medication knowledge can lead to increased utilization of healthcare resources, including emergency department visits, underscoring the clinical importance of this issue (15, 16).

Patients’ ability to understand medication regimens and to use both prescription and over-the-counter medications safely and accurately are strongly influenced by their level of health literacy (17). Several studies suggest that people with substance use disorders typically have low health literacy (18, 19). These individuals are less likely to seek health care when indicated and often fail to adhere to health care recommendations (20). In addition, an association between low health literacy and poor medication adherence has been documented (21).

Despite its obvious clinical importance, patients’ knowledge of their medication is a poorly investigated topic. In fact, to our knowledge, there are no studies that assessed the knowledge of prescribed regular medications, such as withdrawal medications, in patients with various addiction disorders. In the present study, we therefore analyzed the medication knowledge of patients with substance use disorders treated in the psychiatric department of a university hospital in northern Germany. We investigated possible influences of age or gender on medication knowledge and whether patients’ medication knowledge differed between drug groups. This study aims to improve drug and patient safety in the field of addiction psychiatry.

## Methods

### Ethics approval

This study was approved by the Ethics Committee of Hannover Medical School (No. 10762_BO_K_2023) and adhered to the Declaration of Helsinki and its later amendments.

### Study design

The study was designed as a cross-sectional, interview-based study. It was planned to enroll a convenience sample of 100 patients. For this purpose, patients undergoing a detoxification treatment at the Department of Psychiatry, Social Psychiatry, and Psychotherapy of Hannover Medical School between March 2023 and April 2024 were invited to participate in the study. In our clinic, the standard protocol for alcohol detoxification includes oxazepam to prevent alcohol withdrawal seizures, delirium, blood pressure dysregulation, and other withdrawal symptoms, as well as thiamine and a vitamin B complex to prevent Wernicke encephalopathy. Patients willing to participate in the study were informed about the aims of the study, and written informed consent was obtained. Subsequently, patients were screened for eligibility (see section “Eligibility Criteria”). Patients who met all eligibility criteria were interviewed by MD, using a questionnaire specifically adapted to this study (see section “Study questionnaire”). Patients were allowed to use their medication plan during the interview. Based on the median age of the study population (i.e., 46.5 years), we divided the patients into younger (≤ 46 years) and older age groups ≥ 47 years).

### Study questionnaire

In order to address the research questions of this study, the questionnaire by Krause et al. (22) was adapted, excluding the questions on the rules for sick days. The questionnaire used in the present study is available as **Supplementary Material 1** (English version) and **Supplementary Material 2** (German version). The questionnaire was used by MD during the interviews and addressed the following medication-related topics: drug name; indication; dosage; and frequency of application.

These medication-related topics were investigated for each drug in the medication plans of the study participants. One point was achievable for the categories drug name and frequency of application, while up to two points were achievable for the categories indication and dosage. For each drug, the achieved points were summed up to yield the medication knowledge score (range 0-6, with higher scores indicating better medication knowledge). To allow for meaningful comparisons between patients taking different numbers of drugs, the medication knowledge scores of all drugs of a patient were summed up and subsequently divided by the number of drugs the patient was taking, yielding the average medication knowledge score (range 0-6, with higher scores indicating better medication knowledge). Detailed examples of patients’ responses and corresponding ratings can be found in the work by Krause et al. (22). For instance, a patient who answered that she took “Zoloft, against listlessness, one tablet in the morning” would achieve 4 out of 6 points for this drug (a perfect, 6-point answer in this example would have been: “Zoloft, against depression, one 50-mg tablet in the morning”). Correctness of answers was evaluated based on the medication plan from the Department of Psychiatry, Social Psychiatry and Psychotherapy.

In addition, patients’ opinion about their daily number of drugs was evaluated on a 5-point Likert scale: 1 = “too few”; 2 = “rather too few”; 3 = “adequate number”; 4 = “rather too many”; 5 = “too many”.

Finally, patients were asked who or what contributed most to their medication knowledge, using a predefined 9-item list of answer options (single choice): pharmacy; general practitioner; practicing specialist; hospital physician; partner/spouse, relatives, friends; television; the press, magazines; the Internet; or other.

### Occupational position

Occupational positions were classified according to the system proposed by Blossfeld et al. (23). The original classification comprises 12 occupational categories that are homogeneous concerning education, vocational training, and occupational activities. For the purpose of this analysis, these categories were condensed into six groups: unemployed, unskilled (e.g., unskilled service personnel), skilled (e.g., skilled manual occupations such as glassblowers), specialists (e.g., semi-professions such as nurses), highly qualified positions (e.g., professions such as university professors), and others.

### Eligibility criteria

Patients were eligible for enrollment in the study (i) if they were treated at the Department of Psychiatry, Social Psychiatry and Psychotherapy of Hannover Medical School between March 2023 and April 2024; (ii) if they suffered from a substance use disorder; (iii) if they had been regularly taking at least one drug in addition to withdrawal medication; (iv) if they had already been hospitalized for 72 hours and (v) if they did not show cognitive impairment in the clinical examination and were able to provide written informed consent.

### Drug classification and calculation of standard drinks

With regard to our analyses, we differentiated between medication explicitly prescribed for inpatient detoxification treatment and regularly prescribed medication. Drugs were classified according to the Anatomical Therapeutic Chemical (ATC) Classification for Germany, version 2022 (24). For statistical analyses, first-level ATC codes were used.

The calculation of the amount of standard drinks was based on the definition of the World Health Organization (WHO). The WHO guideline regarding brief intervention for risky drinking defines a standard drink as 10 g of pure ethanol (25).

### Statistical analyses

All statistical analyses were performed with IBM SPSS Statistics for Windows, version 28 (Armonk, New York, USA). Quantitative variables were tested for normal distribution with the Shapiro-Wilk test. Due to skewed distribution, quantitative variables are depicted as medians with interquartile ranges (IQRs). For quantitative variables, the Mann-Whitney *U* test or Kruskal-Wallis test was used to investigate potential differences between two groups or ≥ three groups, respectively. Categorical variables are reported as absolute and relative frequencies. Statistical significance was defined as a two-sided p-value < 0.05. The Bonferroni correction was applied to adjust p-values for multiple testing in pairwise comparisons within the Kruskal-Wallis test.

## Results

### Study population and interview characteristics

The study cohort comprised 100 participants with a median age of 46.5 years (IQR 37.8-54; range 20-66), of whom 62% were male (**Table 1**). Participants took a median of 6 medications daily (IQR 5-8; range 1-13). The most prevalent substance-related dependence disorders were alcohol use disorder (89%), cannabis use disorder (21%), and cocaine use disorder (14%). Additional psychiatric diagnoses included depression (53%), personality disorders (23%), and post-traumatic stress disorder (20%). The most common somatic conditions were arterial hypertension (26%), type 2 diabetes mellitus (8%), and chronic obstructive pulmonary disease (7%). Furthermore, 15% of the participants were under legal guardianship. In terms of employment status, 29% were unemployed. The median amount of alcohol consumed in our study population irrespective of the underlying diagnosis was 22 standard drinks per day (IQR 13.5-33.4; range 0-161). The median number of inpatient withdrawal treatments in the past was 4 (IQR 2-15; range 1-60). Three percent of patients used their medication plan as a source of support during the interview.

**Table 1:** Characteristics of the study population (n = 100).

Variables	n	%
Sex		
Female	38	38
Male	62	62
Psychiatric diagnoses^a^		
Alcohol use disorder	89	89
Multiple substance dependence	11	11
Sedative use disorder	9	9
Cannabis use disorder	21	21
Cocaine use disorder	14	14
Opioid use disorder	12	12
Amphetamine use disorder	3	3
Depression^b^	53	53
Schizophrenia or schizophreniform disorder^c^	6	6
Personality disorder^d^	23	23
PTSD	20	20
Amnestic syndrome	2	2
Other psychiatric disorders	45	45
Somatic diagnoses^a^		
Arterial hypertension	26	26
Coronary heart disease	6	6
Chronic heart failure	1	1
Atrial fibrillation	2	2
Status post stroke	2	2
Type 2 diabetes mellitus	8	8
Chronic obstructive pulmonary disease	7	7
Hypothyroidism	3	3
Urinary tract infection	1	1
Other somatic disorder(s)	70	70
Legal factors		
Legal guardianship	15	15
Professional position		
Unskilled	13	13
Skilled	5	5
Specialist	46	46
Highly qualified	4	4
Unemployed	29	29
Other	3	3

^a^Patients could have more than one diagnosis; ^b^ICD-10 F32, F33; ^c^ICD-10 F06.2, F20; ^d^ICD-10 F60; ICD-10 F10.5.

ICD-10, International Statistical Classification of Diseases and Related Health Problems 10^th^ Revision; PTSD, post-traumatic stress disorder.

The median age of the study cohort was 46.5 (IQR 37.8-54) years.

### Average medication knowledge score

The median average medication knowledge score among the study population was 3.8 (IQR 3-4.7; range 0.3-6.0). No statistically significant difference in overall medication knowledge was observed between men and women (median 3.8 (IQR 3-4.7) vs. median 3.7 (IQR 3-4.7); p = 0.679), nor between younger and older patients (median 3.9 (IQR 3.2-4.8) vs. median 3.7 (IQR 2.7-4.6); p = 0.230).

The median score for regular medication knowledge in the study population was significantly different from the median score for withdrawal medication (median 4.8 (IQR 3.6-5.7) vs. median 3.0 (IQR 1.7-4); p = < 0.001). Similarly, no statistically significant differences in regular medication knowledge were found between men and women (median 4.8 (IQR 3.5-5.9) vs. median 4.7 (IQR 3.3-5.4); p = 0.514), nor between younger and older patients (median 4.8 (IQR 3.8-5.7) vs. median 4.8 (IQR 3.1-5.7); p = 0.457).

For withdrawal medication knowledge, there were also no significant differences between men and women (median 3.3 (IQR 2-4.3) vs. median 3.3 (IQR 1.5-4.5); p = 0.858), nor between younger and older patients (median 3.6 (IQR 2.2-4.8) vs. median 3 (IQR 1.6-4); p = 0.418).

### Comparison of drug groups

To investigate potential differences in patients’ medication knowledge across different drug groups, we compared the four most frequently used drug groups in the study population: ATC groups A (alimentary tract and metabolism), B (blood and blood-forming organs), C (cardiovascular system), and N (nervous system) (**Table 2**). The remaining drugs were categorized as “other”, and comprised ATC groups D (dermatologicals), G (genitourinary system and sex hormones), H (systemic hormonal preparations, excluding sex hormones and insulins), J (anti-infectives for systemic use), R (respiratory system), S (sensory organs), and V (various).

**Table 2:** Categorization of drugs (n = 659) taken by the study population according to the World Health Organization’s Anatomical Therapeutic Chemical (ATC) classification system.

Anatomical Therapeutic Chemical (ATC) group (1^st^ level)	n	%
A (Alimentary tract and metabolism)	231	35.1
B (Blood and blood forming organs)	24	3.6
C (Cardiovascular system)	93	14.1
D (Dermatologicals)	4	0.6
G (Genitourinary system and sex hormones)	7	1.1
H (Systemic hormonal preparations, excluding sex hormones and insulins)	7	1.1
J (Antiinfectives for systemic use)	6	0.9
L (Antineoplastic and immunomodulating agents)	2	0.3
M (Musculoskeletal system)	5	0.8
N (Nervous system)	255	38.7
R (Respiratory system)	18	2.7
S (Sensory organs)	2	0.3
V (Various)	5	0.8

Patients’ medication knowledge scores varied significantly between ATC groups A, B, C, N, and the group of “other” medications (global p < 0.001). Medication knowledge scores were significantly lower for ATC group A (median 3 (IQR 0-5)) when compared with ATC group B (median 6 (IQR 4.3-6); p < 0.001), C (median 5 (IQR 3-6); p = 0.005), and N (median 5 (IQR 4-6); p < 0.001) in pairwise comparisons.

### Patients’ opinion about their daily number of drugs to take

More than four-fifths (84%) of the patients considered their daily number of drugs to take “adequate”, while 13% of the patients found that they had to take “rather too many” drugs per day.

### Primary sources of medication knowledge

Forty percent of patients reported receiving most information about their medications directly from their hospital physicians. General practitioners were the second most common source (24%). A smaller portion of the patients (15%) sought information primarily on the Internet, while 14% were mainly informed by practicing specialists such as cardiologists or neurologists. Two percent of patients reported getting most of their information from their spouse or partner. Notably, pharmacies and medication package inserts each accounted for only 1%. None of the patients cited television programs, the press, or magazines as primary sources of information. Additionally, 3% of patients utilized “other” sources for their medication information, and upon further inquiry, all of these patients attributed their knowledge to their own professional expertise.

## Discussion

This study analyzed the medication knowledge of patients with SUDs treated in the psychiatric department of a university hospital in Germany. Medication knowledge is a multidimensional construct for which there is no agreed definition in the literature (26). Furthermore, its operationalization has not yet been standardized, which makes it difficult to compare results (27). Patients’ knowledge of their medication is an under-researched topic. To our knowledge, no studies have investigated knowledge of prescribed medications in patients with SUDs to date. Our study population differed from previous studies in terms of age, gender, and comorbidity profiles. The median age of our study population was 46.5 years, with depression being the most common psychiatric diagnosis besides substance use disorders. The most prevalent substance-related dependence disorders were alcohol use disorder, cannabis use disorder, and cocaine use disorder.

Several previous studies investigated medication knowledge in geriatric patients and found mixed results for medication knowledge in older people (13, 15, 22, 28, 29). Krause et al., using the same methodology as in our study in a geriatric cohort, found an average median medication knowledge score of 5 (22), compared to 3.8 in our study. Their results also showed that women scored higher on medication knowledge than men, in contrast to our investigation in which we did not detect differences in women’s and men’s medication knowledge. Krause and colleagues demonstrated that patients under 80 years of age displayed higher medication knowledge than those over 80 years of age (22). This latter finding cannot be compared meaningfully to our investigation in which participants were considerably younger on average and the cut-off between “younger” and “older” patients was drawn between 46 and 47 years.

In a study by Najjar and colleagues, about four out of five older people recognized the name of the drug, while only 17% could name a side effect of the drug (13). Similarly, Chan et al. reported that 96% of patients were unable to report any possible side effects of the prescribed medication (15). Zwietering and colleagues stated that patients’ reported medication intake matched the number of medications listed in their medical records only about 30% of the time (29). Sancar et al. found that 54.8% of patients surveyed did not know why they were taking their medication and 60.3% did not know when or how to take their medication.

Numerous studies have investigated the knowledge of medication in various patient groups who were not explicitly suffering from a dependency disorder. Some studies suggest that poorer medication knowledge correlates with older age (30–32), a finding which we did not detect in our study. Marvanova et al. reported that lower cognitive function, lower health literacy and higher number of prescribed medications were associated with lower medication knowledge (33). In a cohort of primary care patients, Passagli et al. found that an increased number of comorbidities was associated with poorer medication knowledge (34). In their study, approximately 30% of the patients demonstrated insufficient knowledge regarding their medication (34). In contrast, a study of analgesic prescriptions by Timmerman et al. found that around 50% of participants lacked knowledge about at least one critical aspect of their prescription, such as the drug name, dosage or frequency of administration (35).

It might be speculated that patients undergoing qualified withdrawal treatment in our study generally had lower cognitive functions than patients who did not have an addiction disorder or were not undergoing acute treatment for it, and that this may have been the reason for the lower drug knowledge scores (36). Although we did not carry out extensive neuropsychological testing, cognitive impairment was ruled out as part of the clinical assessment for eligibility.

Strikingly, only 3% of participants in our study used their medication charts during the interview, compared with 95% in the study by Krause and colleagues (22). Freyer et al. found that the use of a medication chart was associated with better medication knowledge (32), which may explain the overall higher average medication knowledge score in the Krause et al. study (22) compared to our investigation.

Patients’ medication knowledge scores were significantly lower for ATC group A when compared to ATC groups B, C, and N. By contrast, in the abovementioned study by Passagli et al. among primary care patients, musculoskeletal drugs were associated with low medication knowledge (34). It is noteworthy that ATC group A includes primarily drugs prescribed during inpatient alcohol withdrawal treatment, such as thiamine, and patients’ knowledge about withdrawal medications was significantly lower compared to their knowledge about regular medication. Substitution of thiamine during withdrawal to prevent Wernicke’s encephalopathy is well-established clinical practice (37). More than four-fifths of the patients considered their daily number of drugs to take adequate, suggesting a high level of trust in prescribers, especially hospital physicians, who also contributed most to patients’ medication knowledge in our study.

In the present investigation, almost half of the patients (46%) were classified as specialists based on their professional position. Higher levels of education and income appear to correlate with better medication knowledge, as suggested by previous research (38, 39). On the other hand, 29% of patients in our cohort were unemployed. Problematic substance use not only increases the likelihood of unemployment, but also reduces the chances of obtaining and maintaining employment (40). Conversely, unemployment is a significant risk factor for harmful use of illicit drugs (40).

Remarkably, in the 21st century, 78% of participants in our study stated that doctors, including general practitioners and specialists, were the main source of their medication knowledge, rather than the media or the internet. In contrast, pharmacists did not appear to contribute to patients’ medication knowledge to a relevant degree in this study.

Although most of the other studies used different methods to assess medication knowledge, which limits direct comparability with our results, it is reasonable to assume that the average medication knowledge of 3.8 out of 6 in our study indicates room for improvement in patient education about medications, especially for ATC group A drugs. This is particularly important since Germany has one of the highest alcohol consumption rates in the world (41). In addition, in light of the recent legalization of cannabis in Germany (42), the potential increase in substance-related dependence requires vigilance (41). Meeting these challenges requires concerted efforts by all healthcare professionals to ensure appropriate pharmacotherapy and comprehensive care for patients with SUDs.

### Strength and limitations

This study has several strengths. First, it addresses a significant gap in the literature by investigating medication knowledge among patients with SUDs, a population that faces unique healthcare challenges and frequent polypharmacy. The structured interview-based approach allowed for a detailed assessment of individual medication knowledge, including nuanced aspects such as drug names, indications, dosages, and administration frequency. Additionally, the study’s findings are clinically relevant, as they highlight specific deficits, particularly concerning ATC group A drugs, providing actionable insights for improving patient education and medication safety in addiction psychiatry. The inclusion of a diverse cohort with varying ages, comorbidities, and substance use patterns further enhances the applicability of the findings to similar clinical populations.

On the other hand, our study also has limitations. The monocentric design, conducted at a single university hospital in Germany, may reduce the generalizability of the results to other geographical or healthcare contexts. The relatively small sample size of 100 participants might have constrained the statistical power to detect more subtle subgroup differences. Additionally, 89% of the study participants suffered from alcohol use disorder, which limits the generalizability of the findings to other SUDs. Although the questionnaire was adapted from a previously used instrument, it was not formally validated for the specific population studied, which could negatively impact the reliability of the results (22).

In conclusion, our study has shown that there is still potential for improvement in the medication knowledge of patients with SUDs, particularly with regard to prescribed medications during qualified withdrawal treatment. Future studies should investigate whether there is a correlation between poor medication knowledge and worse therapeutic outcomes, possibly due to worse adherence to prescribed medicines.

## Data Availability

The raw data supporting the conclusions of this article will be made available by the authors, without undue reservation.
