# Supplementary material for: What do patients with substance use disorders know about their medication? A cross-sectional interview-based study
Source: Front Psychiatry. 2025 Apr 10;16:1556920. doi: 10.3389/fpsyt.2025.1556920 (PMC12018493; doi:10.3389/fpsyt.2025.1556920)
Supplement: Supplementary file 1 [file Table1.docx]

**Questionnaire about medication knowledge of patients with substance use disorders**

**Date: Patient ID: Was a medication plan used during the interview? □ Yes □ No**

| **Drug no.** | What is the name of the drug? | | Is the indication of the drug known? | | | What is the dose of the drug? | | | How frequently is the drug taken? | |
| --- | --- | --- | --- | --- | --- | --- | --- | --- | --- | --- |
|  | 0 | 1 | 0 | 1 | 2 | 0 | 1 | 2 | 0 | 1 |
|  |  | |  | | |  | | |  | |
|  |  | |  | | |  | | |  | |
|  |  | |  | | |  | | |  | |
|  |  | |  | | |  | | |  | |
|  |  | |  | | |  | | |  | |
|  |  | |  | | |  | | |  | |
|  |  | |  | | |  | | |  | |
|  |  | |  | | |  | | |  | |
|  |  | |  | | |  | | |  | |
|  |  | |  | | |  | | |  | |
|  |  | |  | | |  | | |  | |
|  |  | |  | | |  | | |  | |
|  |  | |  | | |  | | |  | |
|  |  | |  | | |  | | |  | |
|  |  | |  | | |  | | |  | |
|  |  | |  | | |  | | |  | |
|  |  | |  | | |  | | |  | |

How would you evaluate the number of drugs you are taking per day?

| 1 = too few | 2 = rather too few | 3 = adequate number | 4 = rather too many | 5 = too many |
| --- | --- | --- | --- | --- |

Who has contributed the most to your medication knowledge/From whom do you receive the most information about your medication? (single choice)

O Pharmacy O Television

O General practitioner O The press, magazines

O Medical specialist O Internet, Apps

O Partner/spouse, relatives, friends O Other

Occupational position?

| O unemployed | O highly qualified positions (e.g. university professorships) |
| --- | --- |
| O unskilled | O other: |
| O skilled (e.g. skilled manual occupations such as glassblowing) |  |
| O Specialized (e.g. semi-professions such as nursing) |  |

Further information?

| O Amount of alcohol consumed: | O other: |
| --- | --- |
| O number of detoxifications: |  |
| O legal support |  |
|  |  |

**Notes during the interview**

________________________________________________________________________________________________________________________________________________________________________________________________________________________________________________________________________________________________________________________________________________________________________________________________________________________________________________________________________________________________________________________________________________________________________________________________________________________________________________________________________________________________________________________
