# Supplementary material for: What do patients with substance use disorders know about their medication? A cross-sectional interview-based study
Source: Front Psychiatry. 2025 Apr 10;16:1556920. doi: 10.3389/fpsyt.2025.1556920 (PMC12018493; doi:10.3389/fpsyt.2025.1556920)
Supplement: Supplementary file 2 [file Table2.docx]

**Fragebogen zum Thema Medikationswissen bei Patient*innen mit Abhängigkeitserkrankungen**

**Datum: Patienten-ID: Wurde ein Medikationsplan zur Beantwortung genutzt?** □ Ja □ Nein

| **Medikament Nr.** | Wie lautet der Name des Medikaments? | | Ist der Verschreibungs­grund bekannt? | | | In welcher Dosis wird das Medikament eingenommen? | | | Wie häufig wird das Medikament eingenommen? | |
| --- | --- | --- | --- | --- | --- | --- | --- | --- | --- | --- |
|  | 0 | 1 | 0 | 1 | 2 | 0 | 1 | 2 | 0 | 1 |
|  |  | |  | | |  | | |  | |
|  |  | |  | | |  | | |  | |
|  |  | |  | | |  | | |  | |
|  |  | |  | | |  | | |  | |
|  |  | |  | | |  | | |  | |
|  |  | |  | | |  | | |  | |
|  |  | |  | | |  | | |  | |
|  |  | |  | | |  | | |  | |
|  |  | |  | | |  | | |  | |
|  |  | |  | | |  | | |  | |
|  |  | |  | | |  | | |  | |
|  |  | |  | | |  | | |  | |
|  |  | |  | | |  | | |  | |
|  |  | |  | | |  | | |  | |
|  |  | |  | | |  | | |  | |
|  |  | |  | | |  | | |  | |
|  |  | |  | | |  | | |  | |

Wie schätzen Sie die Anzahl der von Ihnen eingenommenen Medikamente ein?

| 1 = zu wenige | 2 = eher zu wenige | 3 = angemessene Anzahl | 4 = eher zu viele | 5 = zu viele |
| --- | --- | --- | --- | --- |

Wer trägt am meisten zu Ihrem Wissen über Ihre Medikamente bei bzw. von wem erhalten Sie die meisten Informationen über Ihre Medikamente? (**Einfachauswahl**)

| O Apotheke | O Fernsehprogramm |
| --- | --- |
| O Hausarztpraxis | O Presse, Zeitschriften |
| O Facharztpraxis | O Internet, Apps |
| O Ehepartner, Angehörige, Freunde | O Andere: |

Berufliche Position?

| O erwerbslos | O hochqualifizierte Positionen (z. B. Universitätsprofessuren) |
| --- | --- |
| O ungelernt | O Andere: |
| O qualifiziert (z. B. qualifizierte manuelle Berufe wie Glasbläser) |  |
| O spezialisierte (z. B. Semiprofession wie Krankenpflege) |  |

Weitere Informationen?

| O Alkohol-Konsummenge: | O Andere: |
| --- | --- |
| O Anzahl der Entgiftungen: |  |
| O rechtliche Betreuung |  |
|  |  |

**Notizen während des Interviews**

________________________________________________________________________________________________________________________________________________________________________________________________________________________________________________________________________________________________________________________________________________________________________________________________________________________________________________________________________________________________________________________________________________________________________________________________________________________________________________________________________________________________________________________
